# A study protocol for a cluster randomised controlled trial on mindfulness-based stress reduction: studying effects of mindfulness-based stress reduction and an additional organisational health intervention on mental health and work-related perceptions of teachers in Dutch secondary vocational schools

**DOI:** 10.1186/s13063-020-4189-3

**Published:** 2020-05-04

**Authors:** Math Janssen, Yvonne Heerkens, Beatrice Van der Heijden, Hubert Korzilius, Pascale Peters, Josephine Engels

**Affiliations:** 1 Occupation and Health Research Group, HAN University of Applied Sciences, Nijmegen, the Netherlands; 2 Institute for Management Research, Radboud University, Nijmegen, the Netherlands; 3 Open University of the Netherlands, Heerlen, the Netherlands; 4 Ghent University, Ghent, Belgium; 5 Hubei University, Wuhan, China; 6 Kingston University, London, UK; 7 Nyenrode Business Universiteit, Breukelen, Amsterdam, the Netherlands

**Keywords:** Mindfulness-based stress reduction, Additional organisational health intervention, Mental health, Teachers, Longitudinal approach, Mindfulness, Burnout, Stress, Work engagement, Work performance

## Abstract

**Background:**

Dutch teachers in secondary vocational schools suffer from stress and burnout complaints that can cause considerable problems at work. This paper presents a study design that can be used to evaluate the short-term and long-term effectiveness of mindfulness-based stress reduction (MBSR), a person-focused intervention, both within and outside of the context of an additional organisational health intervention.

**Methods:**

The proposed study comprises a cluster randomised controlled trial that will be conducted in at least three secondary vocational schools, to which teachers will be recruited from three types of courses: Care, Technology, and Economy. The allocation of the intervention programme to the participating schools will be randomised. The teachers from each school will be assigned to intervention group 1 (IG 1), intervention group 2 (IG 2), or the waiting list group (WG). IG 1 will receive MBSR training and IG 2 will receive MBSR training combined with an additional organisational health intervention. WG, that is the control group, will receive MBSR training one year later. The primary outcome variable of the proposed study is mindfulness, which will be measured using the Dutch version of the Five Facet Mindfulness Questionnaire (FFMQ-NL). In the conceptual model, the effects of teachers’ mindfulness resulting from the intervention programmes (MBSR training and MBSR training combined with an additional organisational health intervention) will be related to salient (secondary outcome) variables: mental health outcomes (e.g., burnout, work engagement), work performance, work-related perceptions (job demands and job resources), and personal competencies (e.g., occupational self-efficacy). Data will be collected before (T_0_) and immediately after the MBSR training (T_1_), and 3 (T_2_) and 9 months (T_3_) after the training. The power analysis revealed a required sample size of 66 teachers (22 in each group).

**Discussion:**

The proposed study aims to provide insight into (1) the short-term and long-term effects of MBSR on teachers’ mental health, (2) the possible enhancing effects of the additional organisational health intervention, and (3) the teachers’ experiences with the interventions (working mechanisms, steps in the mindfulness change process). Strengths of this study design are the use of both positive and negative outcomes, the wide range of outcomes, both outcome and process measures, longitudinal data, mixed methods, and an integral approach. Although the proposed study protocol may not address all weaknesses of current studies (e.g., self-selection bias, self-reporting of data, the Hawthorne effect), it is innovative in many ways and can be expected to make important contributions to both the scientific and practical debate on how to beat work-related stress and occupational burnout, and on how to enhance work engagement and work performance.

**Trial registration:**

Dutch Trial Register (www.trialregister.nl): NL5581. Registered on 6 July 2016.

## Background

### Work-related stress and its consequences

Dutch society needs healthy teachers to maintain and improve the quality of the education sector and to enhance student performance [1]. In all sectors, however, work-related stress has become an inherent feature of the employment relationship in industrialised countries such as the Netherlands [2]. Work-related stress is an increasingly important cause of workers’ mental health problems, such as stress symptoms, overstrain, and burnout, which can decrease work performance [3, 4]. In 2017, almost one in six Dutch employees reported stress or burnout complaints. In the educational sector, this figure was more than one in five employees [5]. More than 30% of teachers have reported that major changes in the work context are an important cause of work-related stress. Teachers are expected to meet higher job demands (e.g., high workload, emotional strain) with fewer job resources, especially less professional autonomy [5].

Job demands can be defined as the physical, social, or organisational aspects of the job that require sustained physical or psychological effort [6]. The increase in teachers’ workload is caused by numerous administrative tasks and school reforms. The growing needs of students also generate emotional strain [7]. Job resources can be defined as the physical, social, or organisational aspects that may help teachers to achieve goals and to stimulate learning and development. As such, job resources can buffer the influence of job demands [6, 8].

Work-related stress is associated with several negative organisational outcomes, such as increased absenteeism and early retirement [5]. In comparison with the agriculture, information, and communication sectors, the absenteeism rate in the educational sector is relatively high: 5.3% in the latter versus less than 3% in the former sectors in 2017 [5]. In the Netherlands, the costs of work-related stress absenteeism for the total workforce is €1.8 billion, of which €275 million involves the costs in the educational sector. Work stress-related absenteeism costs are the highest in the educational sector: almost €6000 (number of days × costs per day) for each employee who is absent [9]. When a teacher is absent, organisations in the educational sector strongly rely on the (mostly serendipitous) availability of substitutable colleagues to cover for the absent worker. Consequently, colleagues are overloaded (i.e., a job demand), while the job resources they can draw from remain the same at best. This pattern creates an imbalance between these colleagues’ job demands and resources, which can jeopardise their well-being [7]. This imbalance between job demands and resources and its associated risk of negative effects on one’s well-being may be an important reason that many novice teachers leave the educational sector within the first 5 years of their career [10] and that many experienced teachers retire early. In fact, 45–70% of early retirements in the educational sector can be attributed to psychosomatic and psychological problems [7]. Therefore, it is extremely important to reduce and prevent stress and absenteeism in the occupational sector and to develop effective mental health management interventions, which can be both person-focused and organisation-focused.

### Mental health interventions in the educational sector

A high percentage of Dutch employees (57%), especially in the educational sector, ask for interventions to address work-related stress problems [5]. Many employers in this sector (48%) also recognise the risk of stress [5]. Preventive interventions can be classified as primary, secondary, or tertiary. Primary interventions, which are oriented to the organisational level, aim to change the sources of work-related stress. Secondary and tertiary interventions, both of which are focused on the individual employee, aim to decrease stress symptoms before they cause mental health problems and to treat mental health problems (e.g., burnout), respectively [11]. Mental health interventions in the educational sector are mostly secondary preventive and targeted at the individual level, with the goal of enhancing the ability of teachers to cope with stressors in the workplace [12–18]. Examples are workshops on stress management skills and mindfulness-based stress reduction (MBSR) programmes. MBSR has been shown to be partly effective in influencing mental health outcomes [19].

From a health perspective, primary prevention - when possible - is preferable to secondary and tertiary prevention. In their review of occupational stress interventions in Australia, Caulfield et al. [20] suggested that primary interventions generate more positive changes in comparison with individual-focused secondary or tertiary interventions. However, two meta-analyses on work-related stress interventions [21, 22] found no substantial differences between organisational-level and individual-level interventions. One explanation is the complexity of organisational-level interventions, which might hinder the implementation and measurement of outcomes [7, 23]. In view of this, an appropriate (i.e., mixed-methods) evaluation of an organisational health intervention may require consideration of multiple process outcomes to monitor the implementation process and to investigate the outcomes of the intervention in depth [7, 24]. We agree with Van der Klink et al. [25] that there is a need for an integrated approach that combines both an individual-focused intervention and an organisation-focused intervention.

### Individual-focused secondary health intervention: MBSR

Two systematic reviews have shown that an MBSR intervention programme in the workplace can significantly affect deficit-based outcomes, such as emotional exhaustion (one of the three dimensions of occupational burnout), (occupational) stress, psychological distress, anxiety, and depression [19, 26]. Three systematic reviews also identified significant improvements in asset-based outcomes, such as mindfulness, personal accomplishment (a dimension of burnout), (occupational) self-compassion, quality of sleep, relaxation, and job performance [19, 26, 27]. The systematic review by Donaldson-Feilder et al. [28] reported positive effects on the well-being, resilience, and leadership capability of leaders/managers. Slutsky et al. [29] conducted a randomised controlled trial (RCT) and suggested that small doses of mindfulness training (half-day training) are sufficient to increase job productivity, but that larger doses (6-week training) are needed to improve attentional focus at work, job satisfaction, and work-life balance. The systematic review by Donald et al. [30] identified a positive relationship between mindfulness (both operationalised as a personality variable and as an intervention) and prosocial behaviour.

In a meta-analysis, Klingbeil and Renshaw [31] mentioned that mindfulness-based interventions with teachers are promising for increasing their mindfulness and psychological well-being and for decreasing psychological distress. Overall, they concluded that their findings were similar to the outcomes found in other meta-analyses of the effects of such interventions on employees’ mental health.

Research on mindfulness is often criticised for its poor methodological quality [32, 33]. However, it is impossible to conduct such research using a double-blind placebo-controlled design, which is often applied in medical interventions [34]. It is obvious that participants cannot be kept blind to the fact that they are (or are not) assigned to an MBSR training programme. This raises questions about which methodological features should be included to improve the research design. Goldberg et al. [32] highlighted six features: (1) active control conditions to consider the amount of non-specific attention participants receive, called the Hawthorne effect [35]; (2) larger sample sizes; (3) longer follow-up assessment to measure the sustainability of training effects; (4) evaluation of treatment fidelity; (5) reporting of instructors’ skill levels; and (6) intention-to-treat (ITT) analysis. Three other important features are assessing a diversity of outcomes (negative and positive, process and effect measures, mental health and work performance); using a mixed-methods approach that combines quantitative and qualitative data; and combining an individual-focused intervention, such as MBSR, with an additional organisational intervention (i.e., taking an integrated approach) [19].

### Organisational health interventions

The key points of participatory action research (PAR) [36] are the effective ingredients for organisational interventions: having a bottom-up approach; composing a participatory group; fostering active participation by stakeholders (e.g., employees) and collaboration between researchers and stakeholders; using stakeholders’ knowledge, skills, and perceptions; and creating joint ownership of problems and solutions [7]. Solutions from stakeholders appeared more effective than solutions adopted by others [37]. The belief that one is the master of one’s own behaviour and is able to influence others and the environment (i.e., an internal locus of control) is crucial [38]. In other words, the organisational health intervention should target individuals’ occupational self-efficacy: the belief in one’s own ability in a specific domain of work. The most effective way to enhance one’s self-efficacy is through mastery of experiences [7, 39]. By taking part in the organisational intervention or even by experiencing its effects, we assume that occupational self-efficacy can be influenced to decrease burnout. Indeed, Consiglio et al. [40] found a negative relationship between occupational self-efficacy and burnout, which appeared to be partially mediated by job demands and job resources.

### Aim of the proposed study

The proposed study aims to contribute to the debate on prevention of work-related stress and burnout, and improvement of work engagement and work performance by evaluating the short-term and long-term effectiveness of MBSR, as an individual-focused intervention, on teachers in secondary vocational schools, as an example of a possible application area. It will look at the effects of the intervention on teachers’ mental health (mindfulness as the primary outcome), work performance, work-related perceptions (job demands and job resources), and personal competencies. In addition, it will investigate the effects of a participatory, preventive, organisational health intervention (i.e., a participatory action approach) that targets and engages teachers in a specific course. We hypothesise that participating in the organisational health intervention will positively influence occupational self-efficacy. The application of the organisational health intervention will generate tailored work solutions that may positively influence the balance between job demands (work pressure, work-life balance) and job resources (autonomy, feedback, relationships) for all teachers in schools.

### Conceptual model

**Fig. 1:**
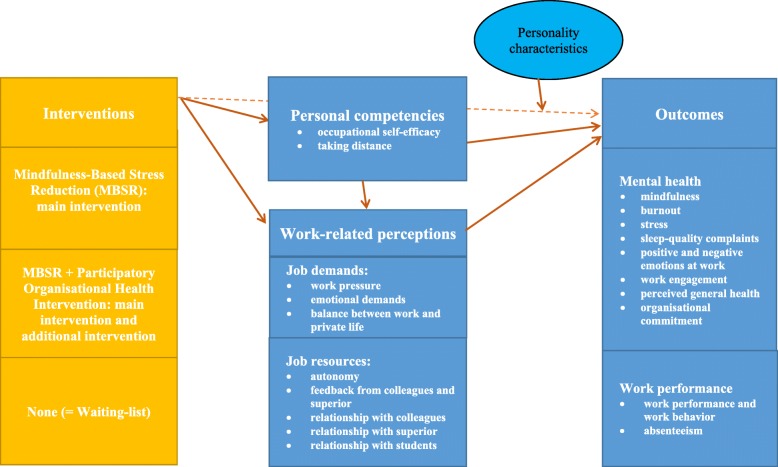
Conceptual model

For this study, we will use a conceptual model (see Fig. 1) inspired by the job demands-resources (JD-R) model [6, 8] and the literature on mindfulness [19, 41–43]. The JD-R model and the literature on mindfulness present two different but complementary points of view on work stress. The original JD-R model has been expanded to include personal resources, aspects of the self, referring to one’s ability to successfully influence the environment. Examples are self-efficacy, emotional stability, extraversion, and resilience [44, 45]. Both the original and expanded model suggest that job characteristics (i.e., job demands and job resources) can influence work stress via two processes. The first process was referred to by Demerouti et al. [6] as the health impairment process, in which high job demands exhaust workers’ mental and physical resources and may therefore lead to a depletion of energy, exhaustion, health problems, and, eventually, premature retirement from their profession. The second process implies a motivational process: job resources have motivational potential that is either intrinsic (because they foster growth, learning, and development) or extrinsic (because they are instrumental in achieving work goals) and lead to positive work outcomes [6]. Job resources and personal resources can buffer the effects of the job demands [6].

Mindfulness, the primary outcome in our study, can reduce stress by separating work characteristics from employees’ reactions to them. This enables the individual to become aware of the difference between observation and interpretation [46].

Figure 1 depicts the conceptual model, including the processes mentioned above and how these relate to mental health outcomes. The right-hand side of the conceptual model presents the study’s secondary outcome variables: mental health outcomes (mindfulness, burnout, stress, sleep quality complaints, positive and negative emotions at work, work engagement, perceived general health, organisational commitment) and work performance outcomes (work performance and work behaviour, absenteeism) [19].

The relationships between the two interventions (MBSR and MBSR with an additional organisational health intervention), on the one hand, and the two clusters of outcome variables, on the other hand, are mediated by two clusters of process variables, which are presented in the middle of the model. The first cluster contains personal competencies that represent the personal resource outcomes resulting from the interventions (occupational self-efficacy, taking distance, as the opposite of worry) [41–43]. The second cluster contains secondary outcome variables: work-related perceptions that refer to how an individual worker experiences work characteristics. In line with the JD-R model, we make a distinction between job demands (work pressure, emotional demands, work-life balance) and job resources (autonomy, feedback from colleagues and superiors, relationship with colleagues, relationship with superiors, relationship with students).

We assume that the five-factor-model of personality, that is the Big Five [47], especially the factors of extraversion and openness, can be expected to positively moderate mental health and work performance. The Big Five consists of five personality characteristics or traits, that are fixed and cannot be developed, in contrast to personal competencies.

## Methods/design

### Study organisation

**Fig. 2:**
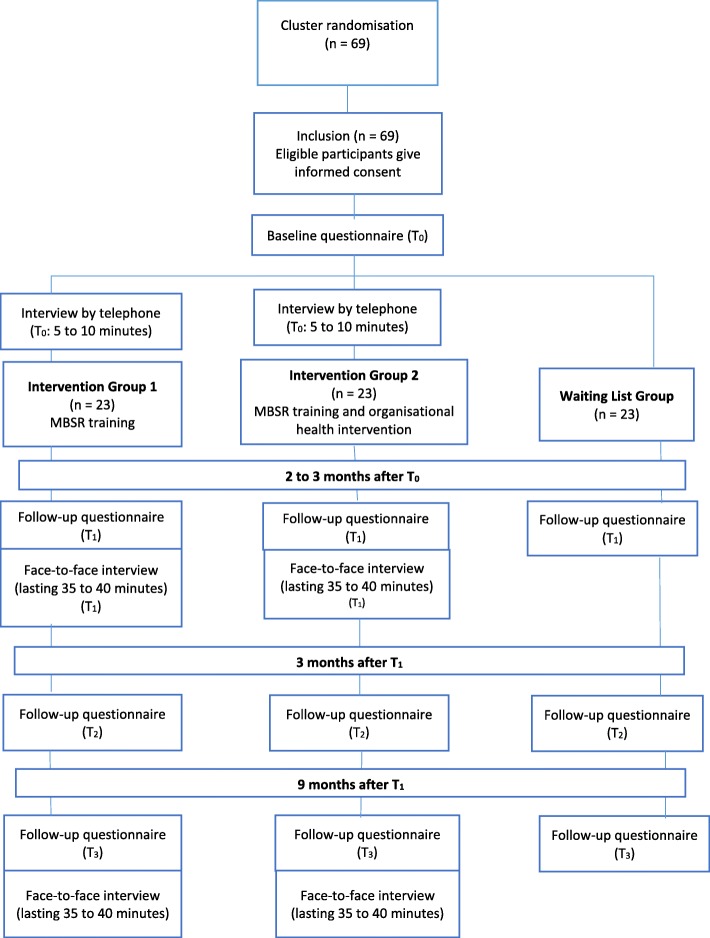
Flowchart showing the design of the trial

The proposed study is a cluster randomised controlled trial (CRCT) that uses a mixed-methods design (quantitative and qualitative, online questionnaire, telephone and face-to-face interviews) and contains four measurement time points (see Fig. 2). The Ethics Committee Practice based Research of het HAN University of Applied Sciences (ECPR) and the Medical Ethics Committee (METC) of Radboud University Medical Centre, both located in Nijmegen, the Netherlands, approved the research proposal (Registration number ACPO 07.12/15; File number CMO: 2019–5266). Both committees stated that the research complied with the requirements of ethical conduct of research as set out in the national Code of Conduct for Scientific Integrity in the Netherlands and that it fulfilled the criteria of the Declaration of Helsinki on Ethical Principles for Medical Research Involving Human Subjects. The study will be carried out in the Netherlands in full compliance with the applicable rules concerning the review of research ethics committees. Participation is voluntary and participants can withdraw at any moment with no consequences. The study title given to the potential participants and other stakeholders is “Mindfulness and job satisfaction of teachers in secondary vocational schools”. Participants will sign informed consent forms before participating in this study. They will be asked if they agree to use of their data should they choose to withdraw from the trial. This trial does not involve collecting biological specimens for storage.

Significant deviations from the protocol will be documented using a breach report form and will be sent to the funder NWO and to the ethics committees. The protocol in the trial register will be updated. A Standard Protocol Items Recommendations for Interventional Trials (SPIRIT 2013) checklist (see Additional file 1) and figure (see Table 1) are provided.

**Table 1:** SPIRIT checklist

	Study period							
	Enrolment		Allocation	Before start	Post-allocation			
TIMEPOINT**		***t***_***0***_			***Intervention***	***t***_***1***_	***t***_***2***_	***t***_***3***_
**ENROLLMENT:**								
**Eligibility screen**	X							
**Informed consent**	X							
***[List other procedures]***	X							
**Allocation**			X					
**INTERVENTIONS:**								
***MBSR training***					X			
***MBSR training and organizational health intervention***					X			
***Waiting list group***								
**DATA COLLECTION** **by on line questionnaire:**								
***Demographics***		X						
***Primary outcome and secondary outcomes***		X				X	X	X
***Other data variables***		X				X	X	X
**DATA COLLECTION** by interviews*:								
***Expectations***		X		X				
***Experiences***						X		X

*SPIRIT* Standard Protocol Items Recommendations for Interventional Trials, *MBSR* mindfulness-based stress reduction

*Interviews were only with some participants in MBSR training and MBSR training and organisational health intervention groups

**T0 = before the training; T1 = immediately after the training; T2 = 3 months after the training; T3 = 9 months after the training

### Participants and recruitment

Study participants will be recruited from the teaching staff at no fewer than three secondary vocational schools. When a secondary vocational school agrees to participate, we will recruit potential participants from three programmes (Care, Technology, and Economy) using e-mail, posters, flyers, and each school’s intranet. The researchers, Human Resources (HR) consultants, and supervisors will inform potential participants about the research project.

Respondents who are willing to participate will be screened in terms of the eligibility criteria by the first author (MJ) (see Table 2). Eligible candidates will receive an information letter about the project. This letter includes the information as approved by the ECPR and the METC and the informed consent letter. One week before the start of the interventions, cluster randomisation will be conducted and the participants will be informed about their assignment to one of the intervention groups or the waiting list group.

**Table 2:** Eligibility criteria

Inclusion criteria	Exclusion criteria
Teachers in the Care, Technology, and Economy courses	Attended mindfulness training in the past 2 years
Employed in a secondary vocational school for at least 2.5 days a week for at least 1 year	Attended stress reduction training (e.g., cognitive therapy or relaxation training) in the past 2 years

### Cluster randomisation

A CRCT is a randomised controlled trial in which groups of subjects (i.e., schools) are randomised rather than individual subjects (i.e., teachers) [48]. Cluster randomisation will be performed at the school level. This will provide the researchers with the opportunity to study the effects of an additional organisational health intervention that cannot be directed towards selected individuals (i.e., teachers) and to control for “contamination” across individuals (i.e., the effects on one teacher may influence the effects on another teacher in the same course) [49]. In the first secondary vocational school (known as an MBO in Dutch), participating teachers from one course (Care, Technology, or Economy) will be assigned to intervention group 1 (IG 1: MBSR), teachers from another course will be assigned to intervention group 2 (IG 2: MBSR and an additional organisational health intervention), and teachers from the third course will be assigned to the waiting list group (WG). The allocation will be different at each school (see Table 3). A researcher who is not involved in assigning courses/participants to the groups will prepare concealed, consecutively numbered, sealed opaque envelopes. Every envelope will contain a paper indicating the treatment assignment at school level (type 1, 2 or 3). The MBO schools will receive their envelopes from a researcher who is unaware of the randomisation sequence. The MBO schools can open the envelope in the presence of the researcher and the researcher will be informed about the treatment assignment.

**Table 3:** Cluster randomisation

	Care	Technology	Economy
**MBO school, type 1**	IG 1^a^	IG 2^b^	WG^c^
**MBO school, type 2**	WG	IG 1	IG 2
**MBO school, type 3**	IG 2	WG	IG 1

^a^ IG 1: intervention group 1 (MBSR)

^b^ IG 2: intervention group 2 (MBSR and an additional organisational health intervention)

^c^ WG: waiting list group (control group that will receive MBSR one year later)

There is little evidence of harmful effects of MBSR [19]. Also, we will recruit participants from a healthy target population, therefore, there will be no special criteria for discontinuing or modifying the allocated interventions. In the case of (serious) adverse events and harms from the intervention, the participant concerned will be referred to an occupational health professional and the project management group, consisting of YH, BVdH, PP and JE, the funder NWO and the ethics committees will be informed. A final decision to terminate the trial will be made by the project management group, deliberating at least every 6 weeks or more, when necessary.

The trial conduct will be audited by an annual evaluation report for the funding organisation NWO. The report is also available for the ethics committees and the project management group, which will discuss the progress of the trial every 6 weeks. YH and JE will be responsible for the daily supervision of the trial. The implementation of the interventions and the data collection will be strictly separated.

### Procedures

All study participants will be asked to complete an online questionnaire on a secured website before the start of the intervention(s) (the starting date of the study is different for each school) (T_0_). After completing the questionnaire, the participating schools will be randomly assigned (type 1, 2, or 3; see Table 3), meaning that participants will take part in IG 1, IG 2, or WG depending on the course where they are working. At T_0_, the first author will conduct 10-min telephone interviews with at least 12 participants from IG 1 and 12 participants from IG 2 about their expectations of the interventions. All participants will receive the other three follow-up questionnaires on a secured website after the MBSR training (T_1_), 3 months later (T_2_), and 9 months after the MBSR training (T_3_). The first author will conduct face-to-face interviews with at least 12 participants from IG 1 and 12 participants from IG 2 at T_1_ and T_3._ At T_1_, some members of the participatory group that will be involved in the organisational health intervention - excluding the teachers participating in IG 2 (e.g., a superior, an HR consultant, and the director of the programme) - will be interviewed about the process and effects of the organisational health intervention.

Participants in IG 1 and IG 2 should attend at least four of the nine MBSR sessions, because Bear et al. [50] revealed that structural changes in perceived stress did not occur until after four MBSR sessions [19]. Participants in WG will attend a MBSR programme one year later.

The collected data will be stored on a secure disk to ensure confidentiality. Not the researcher (MJ) but an independent external organisation, assigning encrypted numbers to the participants, will collect the data. The researcher (MJ) cannot link the numbers and the participants. Only the researcher (MJ), the members of the project management group and a methodologist (HK) will have access to the data.

### Interventions

#### MBSR: main intervention

MBSR, developed by Kabat-Zinn [51], is the most common form of secular mindfulness-based training [52]. MBSR aims to reduce suffering or stress [53] and was originally developed for patients with chronic pain. This training programme is primarily based on Kabat-Zinn’s curriculum [51], but it contains elements of mindfulness-based cognitive therapy (MBCT) [54]: in particular, a 3-min breathing space and psycho-education about the nature of thoughts. The MBSR programme will consist of eight 2.5-h weekly group sessions, each with 4–15 participants per group, homework involving 45 min of daily home exercise 6 days a week, and one day with 7-h of silence. The sessions will be supervised by one of the four recruited, qualified, mindfulness trainers, who will receive a training script. The first session will begin with a short introduction to the programme and meet and greet between participants. Each session will consist of different meditation exercises, enquiry, psycho-education, and a specific theme (see Table 4). At the end of each session, participants will be given homework that will be discussed in the subsequent session.

**Table 4:** Content of MBSR group sessions

Session	Theme	Content of group sessions	Homework
1	Automatic pilot	• Introduction • Raisin-eating exercise • Body scan	• Body scan • Attention to routine activity • Eating one meal mindfully
2	Perceiving clearly	• Body scan • Imagery exercise to demonstrate the relationship between thoughts and feelings • Sitting meditation, paying attention to breathing	• Body scan • Attention to breath • Awareness of pleasant events • Attention to routine activity
3	From doing to being: a mode of doing and a mode of being	• Lying-down yoga exercises • Sitting meditation with a focus on breathing, bodily sensations, sounds • Pleasant events • Seeing exercise to demonstrate the difference between observation and interpretation • Three-minute breathing space (mini-meditation)	• Body scan • Lying-down yoga exercises • Attention to breath • Awareness of unpleasant events
4	Be present	• Three-minute breathing space (mini-meditation) • Standing yoga exercises • Unpleasant events; interrelatedness of bodily sensations, feelings, and thoughts • Sitting meditation with a focus on breathing, bodily sensations, sounds, feelings/ emotions, and thoughts	• Body scan • Standing yoga exercises • Sitting meditation • Three-minute breathing space • Awareness of stress reactions
5	Recognising and allowing what really is: reacting versus responding	• Three-minute breathing space (mini-meditation) • Walking meditation • Sitting meditation with a focus on breathing, bodily sensations, sounds, feelings/ emotions, thoughts, and random attention • Automatic stress reactions and stress response • Mid-term evaluation	• Meditation by choice • Three-minute breathing space • Awareness of difficult situations • Awareness of reactions in difficult situations
6	Mindful communication	• Standing yoga exercises • Sitting meditation with a focus on breathing, bodily sensations, sounds, feelings/ emotions, thoughts, and random attention • Mindful communication exercises	• Meditation by choice • Three-minute breathing space
Day of silence	Deepen mindfulness skills in silence	• Various meditation exercises • Silent lunch and tea break	
7	Taking care of yourself: balance in life	• Standing/lying yoga exercises • Sitting meditation • Communication exercises	• Meditation exercises without CD • Attention to routine activities
8	The rest of your life	• Different exercises • Own menu of mindfulness exercises • Maintaining practice: review of supports • Reflection on training • Saying goodbye	• Further sources of information

*MBSR* Mindfulness-based stress reduction

#### Additional organisational health intervention

**Fig. 3:**
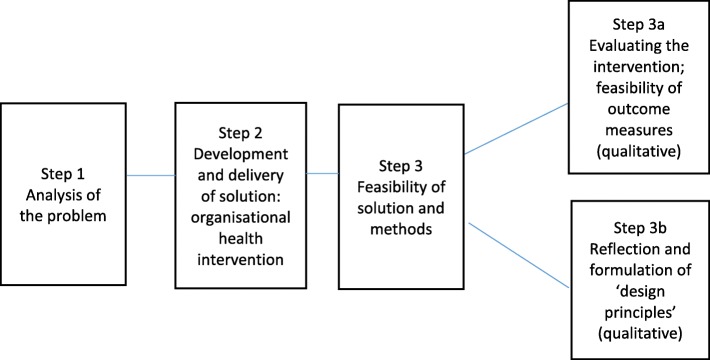
Steps of the design-based approach

The organisational intervention that will be used in the proposed study will be developed following a design-based approach [55] in accordance with the key points of PAR [36] with a grounding in the JD-R model [6]. A design-based approach is pragmatic, based on theory, observations and experiences [56]. The organisational intervention will be developed following the steps of the design-based approach (see Fig. 3). The JD-R model [6, 45] assumes a relationship between work characteristics (i.e., job demands and job resources) and work outcomes. High job-demands lead to stress reactions and unhealthiness (exhaustion process), while high job-resources increase motivation and productivity (motivational process).

The organisational health intervention will consist of two phases. In the first phase, the “needs assessment phase”, we will start with assessment of needs for the implementation of work-oriented solutions, using the knowledge, skills, and perceptions of teachers and educational managers to investigate the positive points (job resources) and the main difficulties (job demands) in the specific course. In the second phase, the “implementation phase”, the teachers and managers will jointly determine the highest priorities and develop a feasible work-related action/implementation plan.

A participatory group will be formed in the needs assessment phase. It will preferably include two teachers participating in the MBSR training, two other teachers (workplace), the HR consultant for the specific course (expert), a supervisor, the course director (decision-making power), an external facilitator, and relevant others from the workplace (e.g., a union member or a member of the formal employee participation committee).

The focus of the intervention is to stimulate dialogue between management and employees/teachers in which they can jointly investigate improvement opportunities and implement solutions that reduce stress and improve work pleasure. The intervention consists of approximately five sessions (see Table 5), starting with assessment of needs to the implementation of work-oriented solutions.

**Table 5:** Sessions and content of participatory group sessions

Session	Content of participatory group sessions	Phase
1	• Introduction • Mutual expectations, drive and mission/vision. Mutual commitment • How to engage colleagues? • First inventory of positive points (job resources) and difficulties (job demands) for work pleasure in the course, as inspired by the JD-R model (Prioritising based on importance, level of influence, and the wait time for results)	1
2	• Which priorities do we choose? • Possible solutions • Action plan • Implementation plan	1
3	• Follow up	2
4	• Follow up	2
5	• Follow-up implementation • Maintaining, continuation, evaluation • Saying goodbye to the external facilitator	2

The programme theory or critical assumptions underpinning the organisational health intervention (*How would the intervention work if it were successfully implemented?*) [57] are as follows:

1. Involving an external facilitator (an expert in organisational change processes) and creating a participatory group that includes teachers, the HR consultant, the director, and the managers will enable the group to establish the highest priorities and develop solutions to improve the working environment.
2. A supported, basic work-related action plan will be developed as well, consisting of: “relatively easy and quick to solve” priorities (= quick wins), solutions, a timeline, necessary resources, and an implementation method.
3. The quick wins will be implemented immediately.
4. Healthy working in the course will be improved, dialogue between management and employees/teachers will be stimulated, and occupational self-efficacy will be increased.

#### Waiting list group

The participants on the waiting list will receive MBSR after one year. They cannot attend a mindfulness training course or stress reduction training (e.g., cognitive therapy or relaxation training) until T_3_.

### Baseline characteristics of participants

Measures of the baseline characteristics of the participants are gender, age (years), family situation, level of education, nature of employment (course Care, Technology, or Economy), years of work experience, and number of working days and hours.

### Baseline characteristics of participants at T_0_

- Gender
- Age (years)
- Family situation
- Level of education
- Nature of employment; course (Care, Technology, or Economy)
- Years of work experience
- Number of working days and hours

### Outcome assessments and data collection

#### Primary outcome

Mindfulness skills will be examined using the Dutch version of the Five Facet Mindfulness Questionnaire (FFMQ-NL), a self-report measure based on a factor analysis of items from the five most widely used mindfulness questionnaires [58, 59]. The 39-item FFMQ-NL has a five-factor structure that is captured in the following five subscales: observing, describing, acting with awareness, non-judging of inner experience, and non-reactivity to inner experience. The FFMQ-NL total score ranges from 39 to 195; the total scores of the subscales are 8–40, except for non-reactivity (7–35). Higher values indicate higher levels of mindfulness skills. De Bruin et al. [59] reported internal consistency of 0.85 (Cronbach’s alpha) for the FFMQ-NL total score (for the non-meditating sample) and 0.90 (for the meditating sample); Cronbach’s alpha for the five subscales varies from 0.70 to 0.89 [59]. There is modest but significant correlation between the five dimensions (ranging from 0.13 to 0.39), which suggests that they represent distinct but interrelated constructs [59]. All mindfulness dimensions are positively correlated with meditation experience and negatively correlated with psychological symptoms (i.e., depression, anxiety, insomnia, and social withdrawal) [59]. All the dimensions except for *observing* are negatively related to the constructs of alexithymia (lack of ability to identify and describe feelings, and lack of interest in feelings, cognitions, and motivations), thought suppression, rumination, worry and dissociation [59]. Overall, the psychometric properties of the FFMQ-NL [60] are comparable to those of the original English version [58].

#### Secondary outcomes

##### Secondary mental health outcomes

Burnout will be measured using the Dutch version of the Maslach Burnout Inventory - Education Survey (MBI-ES), the Utrechtse BurnOut Schaal-Leerkrachten (UBOS-L; Utrecht Burnout Scale - Education) [60–62]. The 22-item UBOS-L has a three-dimensional structure with the following subscales: emotional exhaustion, mental distance (cynicism, depersonalisation), and (job-related) personal accomplishment/professional efficacy. The total scores of the three subscales range from 0 to 6. Higher values indicate more emotional exhaustion, more mental distance, and more personal accomplishment, respectively. Maslach et al. [61] reported Cronbach’s alpha for the three subscales - emotional exhaustion (8 items), mental distance (7 items), and professional efficacy (7 items) - of 0.91, 0.73, and 0.85, respectively. The emotional exhaustion subscale is highly correlated with other mental and physical complaints, and with job demands like time pressure [61]. Mental distance and professional efficacy are significantly related to personal resources like autonomy and ambition level [61].

Stress will be assessed using the 14-item stress scale of the Dutch 42-item Depression, Anxiety, Stress Scales (DASS) [63]. The total score on the stress scale ranges from 0 to 21. Higher values indicate more stress. The DASS has a three-factor structure: depression, anxiety, and stress. Nieuwenhuijsen et al. [63] reported internal consistency of the DASS of 0.94, 0.88, and 0.93, respectively.

Sleep quality complaints will be measured using the Dutch sleep quality subscale of the 14-item Vragenlijst Beleving en Beoordeling van de Arbeid 2.0 (VBBA2.0; Perception and Assessment of Labour 2.0 Questionnaire). The total score ranges from 0 to 100. Higher values indicate more complaints and lower-quality sleep. Van Veldhoven et al. [64] reported internal consistency (Cronbach’s alpha) of 0.90.

Positive and negative emotions at work will be assessed by the 12-item Dutch version of the Job-related Affective Well-Being Scale (JAWS) [65, 66]. The Dutch JAWS has a two-factor structure, which is reflected in the following two subscales: a positive six-item emotions scale (Cronbach’s alpha = 0.77) and a negative six-item emotions scale (Cronbach’s alpha = 0.78) [66]. The total score on each subscale varies from 6 to 30. Higher values indicate more positive emotions and more negative emotions, respectively. The positive emotions subscale is negatively correlated with the frequency (*r* = − 0.22) and duration (*r* = − 0.23) of future absenteeism of managers; the negative emotions subscale is not correlated with these variables [66].

Work engagement will be assessed using the nine-item Dutch version of the shortened Utrecht Work Engagement Scale (UWES), the UBES-9 [67, 68]. The three-dimensional UWES consists of three 3-item subscales: vigour, dedication, and absorption. The total score on the UWES ranges from 9 to 54. Higher values indicate more work engagement. Schaufeli et al. [67] reported internal consistency (Cronbach’s alpha) for the total UBES-9 of 0.93 and the alpha for the three subscales varies from 0.79 to 0.89. The three work engagement scales are highly correlated (minimum *r* = 0.65) [67]. The three factors are negatively correlated with the three dimensions of burnout [67].

Perceived general health will be measured using two items (1 and 11) from the Dutch version of the Short Form 36 Health Survey, version 2 (SF-36-v2), named RAND-36 [69]. The score on each item ranges from 1 to 5; the transformed overall score on the two items varies from 0 to 100. Higher values indicate higher levels of perceived general health. The internal consistency (Cronbach’s alpha) as reported by van der Zee et al. [69] is 0.81.

Organisational commitment will be assessed by four items derived from the four-item Affective Commitment Scale (ACS) used by Smeek et al. [70], who reported reported Cronbach’s alpha of 0.70 for this scale.

##### Secondary work performance outcomes

Work performance and work behaviour*,* defined as behaviours or actions of employees that are relevant to the organisation’s goals, will be measured using the Dutch Individuele WerkPrestatie Vragenlijst (IWPQ; Individual Work Performance Questionnaire) [71]. The 18-item questionnaire consists of three subscales: task performance (5 items), contextual performance (8 items), and counter-productive work behaviour (5 items). The total scores on the three subscales range from 0 to 4. Higher values indicate more task performance, more contextual performance, and more counterproductive work behaviour. The internal consistency (Cronbach’s alpha), reported by Koopmans et al. [71], varies between 0.78 (task performance) and 0.85 (contextual performance). Task performance and contextual performance are moderately positively correlated with work engagement: r values 0.32 and 0.43, respectively. Counterproductive work behaviour is moderately negatively correlated with work engagement (r value − 0.29) [71].

Absenteeism, working fewer than the normal hours or days in the employment contract due to a health problem, will be measured by four items from the NEA 2018 [72], the Dutch Working Conditions Survey 2018 (e.g., *How many working days have you been absent in the last three months? How many times have you been absent in the last 12 months over one or more periods longer than 2 weeks? If so, has the absenteeism to do with your work? Have you fully returned to work now?*).

#### Mediating variables

##### “Personal competencies outcomes” or “process-focused outcome measures”

Occupational self-efficacy, which refers to the confidence a worker has in their perceived ability to perform job tasks successfully, will be assessed using the short (six-item) Dutch version of the Occupational Self-Efficacy Scale [73]. The total mean score ranges from 1 to 6. High values reflect high occupational self-efficacy. Rigotti et al. [73] reported internal consistency (Cronbach’s alpha) of 0.85.

Taking distance, which comprises not worrying or ruminating about work at home, will be assessed using the three-item “Afstand Nem”’ (Taking Distance) subscale of the VBBA 2.0. The total score varies from 0 to 100. Higher values indicate that the individual experiences more problems with taking a distance from work. Van Veldhoven et al. [64] reported internal consistency (Cronbach’s alpha) of the subscale of 0.80.

##### “Work-related perceptions”

The job demands of work pressure and emotional demands will be measured using the six-item Werktempo & Werkhoeveelheid (Work Pace and Workload) questionnaire and the five-item Emotionele Belasting (Emotional Demands) questionnaire of the VBBA 2.0, respectively. The total score ranges from 0 to 100. Higher values indicate more work pressure and more emotional demands. Van Veldhoven et al. [64] reported an internal consistency (Cronbach’s alpha) of the subscales of 0.86 and 0.80, respectively.

The job demand of balance between work and private life will be assessed using two subscales of the Dutch version of the Survey Work-home Interaction-NijmeGen (SWING): the negative Work-Home Interaction (negative WHI) subscale, which measures negative effects of work on functioning at home and the negative Home-Work interaction (negative HWI) subscale, which measures negative effects of home on functioning at work [74]. The SWING also includes two other subscales: the positive Work-Home Interaction (positive WHI) subscale and the positive Home-Work interaction (positive HWI) subscale. The total score on the negative WHI and the negative HWI ranges from 0 to 3. Higher values indicate more problems in work-home interaction. The internal consistency (Cronbach’s alpha) of the negative HWI and the negative WHI, as reported by Geurts et al., is 0.72 and 0.85, respectively [74].

The job resources of autonomy (4 items), feedback from colleagues and superior (4 items)*,* relationship with colleagues (6 items), relationship with superior (6 items), and relationship with students (4 items) will be measured using several scales of the VBBA2.0. The total score on every scale ranges from 0 to 100. Higher values indicate more problems in the specific outcomes (e.g., a higher score on autonomy indicates less autonomy). The internal consistency (Cronbach’s alpha) of the subscales, reported by van Veldhoven et al. [64], varies from 0.81 to 0.87.

#### Moderating variable “personality characteristics”

The Dutch version of the Ten Item Personality Inventory (TIPI) will be used to measure the dimensions of the five-factor-model of personality: neuroticism, extraversion, openness, agreeableness, and conscientiousness. Each factor will be assessed by two unipolar items with a 7-point Likert scale ranging from 1 = not applicable at all to 7 = completely applicable. The TIPI has been shown to be a valid alternative for the existing extensive Big Five instruments [47].

### Process evaluation of the MBSR training

A process evaluation will be conducted to explore working mechanisms and possible barriers to MBSR in this population. The process evaluation of MBSR will be conducted using both quantitative (online questionnaire, primarily questions about experiences with the MBSR training) and qualitative measurements (semi-structured interviews). All participants will receive the online questionnaire at T_0_, T_1_, T_2_, and T_3_. A selection of the participants in IG 1 and IG 2 will be interviewed at T_0_, T_1_, and T_3_. The interview at T_0_, lasting 10 min, will be conducted by telephone and will be focused on expectations about MBSR. The face-to-face interview at T_1_, lasting 25– 35 min, will be about experiences during the MBSR training and its short-term effects. The face-to-face interview at T_3_, lasting approximately 25–35 min, will be focused on long-term effects. All interviews will be recorded, fully transcribed, and anonymised. A deductive qualitative analysis will be performed, because of the availability of a focused main research question and a conceptual model [75, 76]. The interviews can provide valuable information about the working mechanisms and possible barriers of the MBSR training.

### Process evaluation of the additional organisational health intervention

A process evaluation of the additional organisational health intervention will be performed to assess the requirements/conditions for successful implementation, based on a simplified version of the theoretical framework presented by Nielsen and Randall [57, 77]. These researchers indicate that a process evaluation is important because the implementation process can moderate or mediate the potential effects of the intervention on health and well-being [57, 77]. Successful implementation is a prerequisite for exposure to the intervention and therefore for entailing possible health effects. The framework, which enables us to link intervention processes to intervention outcomes, will be applied to qualitatively appraise 3 themes of process components: (1) intervention design and implementation, determining the maximum level of intervention exposure; (2) intervention context; and (3) participants’ mental models [57, 77]. The process components of themes 2 and 3 may mediate or moderate the link between any intervention exposure and intervention effects [77]. Table 6 lists the themes and requirements/process components for successful implementation that will be assessed in the semi-structured interviews (T_1_). Applying the framework will help us to understand why the implementation process was successful or not [57].

**Table 6:** Themes and requirements/process components for successful implementation, based on a simplified version of the theoretical framework from Nielsen and Randall [77]

Themes and requirements	Operationalisation
1) **Intervention design and implementation**	
Initiation	Commitment to the intervention and the motivation of the director and team managers
Communication about the intervention at the start	Communication to the teachers from the course, the mindfulness training participants, and the participatory group members
Participation	• Establishment of a participatory group • Involvement of the teachers in the course and of the participants in the mindfulness training and in the participatory group
Targeting	Choosing the right problems in the workplace with the possibility of quick wins
Satisfaction	The teachers’/participants’ satisfaction with the intervention
2) **Intervention context**	
Organisation’s culture	Inherent features of the organisation’s culture that facilitate or impede the implementation of the action plan
Conditions	The organisation’s capacity and skills to implement the action plan
Events	Events that interfere with implementation of the action plan
3) **Participants’ mental models**	
Readiness to change	Employees’ and participants’ readiness to change at T_1_
Perceptions	Was the perception of the intervention (action plan) positive?

*T1* timepoint 1 (immediately after mindfulnesss-based stress reduction training)

The process evaluation will be conducted using semi-structured interviews. A selection of participants in IG 2 (taking into account participating in the participatory group or not) and of other participatory group members who are not participating in the MBSR training (e.g., teachers not participating in the MBSR training, or supervisor, director, HR consultant, work council member, trade union member) will be interviewed at T_0_, T_1_, and T_3_.

### Sample size

A power analysis (G*Power; version 3.9.1.4) revealed that a sample size of 22 participants in each group (IG 1; IG 2; WG), with at least two repeated measurements would enable detection of a medium effect size (*d* = 0.50) [78], with power of 0.95 and alpha of 0.05. A total sample size of 66 participants is therefore required.

### Blinding

Participants, trainers, facilitator, and researchers cannot be blinded to their assigned intervention after cluster randomisation. All participants have to fill in the online questionnaire at home or at work, excluding the influence of the researcher. The developer of the online questionnaire will collect the data and provide the anonymous data to the researcher. The researcher will analyse the data blinded to the assigned intervention.

### Statistical analyses

Baseline characteristics of participants will be presented as means and standard deviations (SDs) for metric variables, and as frequencies and percentages for categorical variables. The outcomes of the questionnaires will be compared at baseline (T_0_), immediately after the intervention(s) (T_1_), 3 months later (T_2_), and 9 months after the intervention(s) (T_3_). All analyses will be conducted according to the intention-to-treat (ITT) principle. ITT analysis, based on the initial treatment allocation and not on the treatment eventually received, will avoid the effects of drop-out, and as such we prevent breaking the random allocation to the intervention groups [79]. Per-protocol (PP) analyses in the treatment-adherent sample (i.e., participants in IG 1 and IG 2 have to attend at least four of the nine MBSR sessions, and participants in WG cannot attend a MBSR programme or stress reduction training) will also be performed. The aim of PP analysis is to assess the effects of MBSR and the additional organisational health intervention *under optimal conditions***:** what is the effect if participants are fully compliant [80]? Therefore, drop-outs need to be excluded from any PP analysis.

The quantitative short-term and long-term effects of MBSR and the additional organisational health intervention (differences between T_0,_ T_1_, T_2_, and T_3_) will be examined using longitudinal regression analysis (generalised estimating equations, GEE, or mixed models), which is fit to analyse longitudinal/clustered data in clinical trials [81] or repeated-measures designs (general linear model, GLM) [82]. The baseline values of outcomes (T_0_) of the three groups (IG 1; IG 2; WG) will be defined as independent variables, while the outcomes on the follow-up measurements (T_1_, T_2_, T_3_) will be treated as dependent variables. Correction of confounding variables will be applied.

To investigate the working mechanisms (*How is mental health improved?*) of MBSR and the organisational health intervention, mediating and moderating analyses will be conducted. The mediating effect of personal competencies on mental health outcomes and on work performance outcomes will be tested. The mediating effect of work-related perceptions on mental health outcomes and on work performance outcomes will also be investigated. The moderating effect of the Big Five, especially as regards the factors of extraversion and openness, on mental health outcomes/work performance outcomes will also be examined.

All statistical analyses will be conducted using IBM SPSS Statistics, version 25. The level of significance will be set at 0.05. The analysis of the qualitative data, collected by the semi-structured interviews at T_0_, T_1_, and T_3_, will be deductive [− 76], and will be conducted by means of using ATLAS.ti [83].

### Dissemination policy

Results of the trial will be communicated by scientific articles in open access journals, management letters for participants and non-participants of Dutch secondary vocational schools, and articles for professional magazines intended for occupational health professionals.

## Discussion

The proposed study will evaluate the short-term (T_1_) and long-term effects (T_2_ and T_3_) of mindfulness-based stress reduction (MBSR), a person-focused intervention aimed at strengthening the individual capacity of teachers in secondary vocational schools to cope with stress and enhance their mental health. In addition, the possible enhancing effects of an additional organisational health intervention, a participatory action approach, will be investigated as well. The teachers’ experiences with the interventions (the working mechanisms of MBSR and the organisational health intervention) will also be examined. This study is a cluster randomised controlled trial, in which intervention group 1 (IG 1; receiving MBSR) and intervention group 2 (IG 2; receiving MBSR and an additional organisational health intervention) will be compared with the waiting list group (WG; the control group).

Many previous studies on the effects of MBSR on employees have primarily assessed negative outcomes, focusing predominantly on mental health (e.g., burnout, stress level, psychological distress). Process measures, which are suitable for investigating how mindfulness can contribute to well-being, have rarely been assessed [19]. The strength of this study lies in the fact that we will assess both negative and positive outcomes. More specifically, it considers not only mental health (e.g., positive emotions at work, work engagement, organisational commitment), but also work performance and work-related perceptions (e.g., job demands and job resources). Process measures (e.g., occupational self-efficacy, taking distance) will also be examined. Hence a wide range of outcomes will be measured [19].

In their systematic review on MBSR and employees’ mental health, Janssen et al. [19] reported that 14 of the 23 studies included in the review only incorporated short-term effects, measured immediately after the intervention. However, in terms of the cost-benefit ratio, MBSR and the organisational health intervention should lead to sustainable long-term effects. Therefore, another strength of this study is that it will gather longitudinal data by measuring both short-term and long-term effects (until 9 months after the intervention).

The proposed study will use a mixed-methods approach, which is rare in studies on the effects of MBSR [19]. That approach means that, in addition to quantitative data, qualitative data will be collected to investigate in-depth relevant process measures and to capture the mechanisms by which MBSR (key aspects of the MBSR programme) and the organisational health intervention (factors for successful implementation in an organisation) result in specific outcomes. Another strength of the proposed study is the integrated approach, which combines an individual-focused secondary intervention (MBSR) and an organisation-based primary intervention. This is important since teachers’ stress likely results from a complex interaction between personal characteristics of the teacher and the environment (work and personal circumstances) [6, 84].

The design of the cluster randomised controlled trial (CRCT), in which schools are randomised as opposed to individual teachers, is another strength of our proposed approach. CRCT allows us to study the effects of an organisational health intervention and to control for “contamination” across participants [48, 49].

Despite the many strengths of the proposed study, complying with research ethics implies that we cannot account for some limitations that have already been raised in previous literature. For example, the proposed study design will be based on self-selection, as we depend on voluntary participation by teachers, which may result in somewhat biased samples. Moreover, it is likely that the characteristics of the teachers who participate in the proposed study (e.g., motivation, sensitivity to the MBSR training and the organisational health intervention, personality) may differ from those who will not participate or those who drop out early.

We assume that many participants experience work pressure, time pressure, and stress complaints. The MBSR training (and the additional organisational participatory health intervention) requires a lot of time and effort from the teachers. We are therefore aware that (potential) participants have to be motivated to prevent premature drop-out.

In line with this, the time frame of the organisational health intervention (8–12 weeks) is a short period with regards to capturing organisational changes. Therefore, the T_1_ measurement might occur too soon to detect effects. However, the measurements at T_2_ and T_3_ overcome this limitation.

Data from questionnaires using self-reports may be biased [85]. The primatologist and psychologist De Waal [86] posited that human beings are insufficiently aware of their inner state and may therefore mislead themselves and others. The study of the human psyche needs behavioural reports, based on observation by others [86]. The proposed study will address this concern partly by using both validated questionnaires and data triangulation (both quantitative and qualitative data).

Another potential source of bias is associated with the effect of attention received by teachers in IG 1 and IG 2, also known as the Hawthorne effect [35]. Participating in group sessions in IG 1 and IG 2 may lead to an overestimation of the effect of MBSR and the organisational health intervention. Participants cannot be blinded to the allocated intervention, so the Hawthorne effect cannot be excluded.

The proposed CRCT has some disadvantages compared to an RCT [87]. A CRCT has greater complexity in design and analysis and requires more participants/teachers, to achieve adequate statistical power.

### Trial status

The trial is funded for 5 years. The first participants were randomly assigned in September 2016. Final outcome assessments will be completed in June 2020. This is the first protocol version (31 August 2015).

## Supplementary information

**Additional file 1.** SPIRIT 2013 Checklist: Recommended items to address in a clinical trial protocol and related documents.

## Data Availability

Other than the authors no other entities have contractual agreements with regard to access to the final dataset. The datasets generated and/or analysed during the current study are not publicly available due to the ongoing research, but are available from the corresponding author on reasonable request.

## Abbreviations

ACS: Affective Commitment Scale
CRCT: Cluster randomised controlled trial
DASS: Depression, Anxiety, Stress Scales
ECPR: Ethics Committee Practice-based Research of het HAN University of Applied Sciences, Nijmegen
FFMQ-NL: Five Facet Mindfulness Questionnaire
GEE: Generalised estimating equations
GLM: General linear model
HR: Human resources
HWI: Home-work interaction
IG 1: Intervention group 1
IG 2: Intervention group 2
ITT: Intention-to-treat analysis
IWPQ: Individual Work Performance Questionnaire
JAWS: Job-related Affective Well-being Scale
JDR: Job demands-resources
MBCT: Mindfulness-based cognitive therapy
MBI-ES: Maslach Burnout Inventory - Education Survey
MBSR: Mindfulness-based stress reduction
METC: Medical Ethics Committee
METC: Medical Ethics Committee of Radboud University Medical Centre, Nijmegen
PAR: Participatory action approach
PP: Per-protocol analysis
SDs: Standard deviations
SF-36v2: Short Form 36 Health Survey, version 2
SWING: Survey Work-home Interaction-NijmeGen
TIPI: Ten Item Personality Inventory
UBOS-L: Utrechtse BurnOut Schaal-Leerkrachten (Utrecht Burnout Scale - Education)
UWES: Utrecht Work Engagement Scale (Dutch; UBES)
VBBA2.0: Vragenlijst Beleving en Beoordeling van de Arbeid 2.0 (Perception and Assessment of Labour 2.0)
WG: Waiting list group
WHI: Work-home interaction
